# Supplementary material for: Interhelical H-Bonds Modulate the Activity of a Polytopic Transmembrane Kinase
Source: Biomolecules. 2021 Jun 25;11(7):938. doi: 10.3390/biom11070938 (PMC8301901; doi:10.3390/biom11070938)
Supplement: Supplementary file 1 [file biomolecules-11-00938-s001.zip › biomolecules-1254467-supplementary.pdf]

biomolecules

MDPI

Table S1. Oligonucleotide primers used in this work. The mutations introduced are in minuscule.

TM1	MIKNHFTFQKLNGITPYIWTIFFILPFYFI
TM1-Y17A	MIKNHFTFQKLNGITP**A**IWTIFFILPFYFI
TM1-T20A	MIKNHFTFQKLNGITPYIW**A**IFFILPFYFI
TM1-Y28A	MIKNHFTFQKLNGITPYIWTIFFILPF**A**FI
TM5	MVLKKEFFLTQIPFVVITLISAILLPFSIKS
TM5-T140A	MVLKKEFFLTQIPFVVI**A**LISAILLPFSIKS
TM5-S143A	MVLKKEFFLTQIPFVVITLI**A**AILLPFSIKS
TM5-S150A	MVLKKEFFLTQIPFVVITLISAILLPF**A**IKS

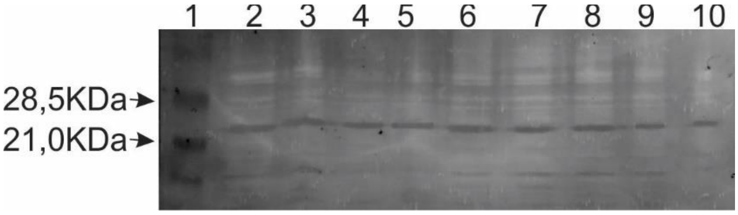

Figure S1. Western blots of the membrane fraction shows that the levels of expression and integration of each Coexp TM1/TM5 are similar: Molecular Weight Ladder (lane 1), Coexp TM1 H188V/TM5 (lane 2), Coexp TM1/TM5 H188V (lane 3), Coexp TM1/TM5 (lane 4), Coexp TM1/TM5 (lane 5), Coexp TM1 Y17A /TM5 (lane 6), Coexp TM1 T20A/TM5 (lane 6), Coexp TM1 Y28A/TM5 (lane 7), Co-exp TM1/TM5 T140A (lane 8), Coexp TM1/TM5 S143A (lane 9), Coexp TM1/TM5 S150A (lane 10).
